# Learning the language of pathogens

**DOI:** 10.7554/eLife.89264

**Published:** 2023-06-15

**Authors:** Izadora Volpato Rossi, Marcel Ivan Ramirez

**Affiliations:** 1 Graduate Program in Cell and Molecular Biology, Federal University of Paraná Curitiba Brazil; 2 Carlos Chagas Institute, FIOCRUZ Curitiba Brazil

**Keywords:** parasite, communication, vesicles, Trichomonas, filopodia, pathogenesis, Other

## Abstract

Parasites can use extracellular vesicles and cellular projections called cytonemes to communicate with one another.

**Related research article** Salas N, Blasco Pedreros M, Dos Santos Melo T, Maguire VG, Sha J, Wohlschlegel JA, Pereira-Neves A, de Miguel N. 2023. Role of cytoneme structures and extracellular vesicles in *Trichomonas vaginalis* parasite-parasite communication. *eLife* **12**:e86067. doi: 10.7554/eLife.86067.

To survive and replicate, pathogens must evade the immune system of the organisms they infect by adapting to the environment inside them. The individual pathogens that manage to do this will survive and multiply, and particularly successful adaptations may lead to the emergence of new strains of the pathogen (Bashey, 2015; Mideo, 2009). Understanding how this happens – including how pathogens communicate with each other, and how they respond to the microbiota of their host (Kalia et al., 2020) – is crucial to efforts to combat the diseases caused by parasites and other pathogens.

Decades of studying host-pathogen interactions has revealed several mechanisms of pathogen virulence, such as variation of surface antigens to avoid immune detection and the release of molecules that neutralize the host immune system and promote pathogen invasion (Casadevall and Pirofski, 2001). Specific structures and organelles that pathogens employ – such as flagella to allow swimming – have also been discovered (Khan and Scholey, 2018).

Cells have been shown to communicate with one another by exchanging membrane-bound ‘extracellular vesicles’ – which contain proteins, lipids and genetic material – with neighbouring cells (van Niel et al., 2018; Raposo and Stahl, 2019). Cells can also communicate via plasma membrane protrusions called ‘filopodia’ which contain bundles of actin filaments and have roles in cellular adherence and migration (Roy and Kornberg, 2015). There are multiple types of filopodia, categorized by their size and origin.

Now, in eLife, Natalia de Miguel (Instituto Tecnológico Chascomús and Escuela de Bio y Nanotecnologías) and colleagues – including Nehuén Salas and Manuela Blasco Pedreros as joint first authors – report that different strains of the protozoan *Trichomonas vaginalis*, a parasite responsible for sexually-transmitted infections, communicate using extracellular vesicles and a type of filopodia called a cytoneme (Salas et al., 2023). Cytonemes are thin specialized filopodia that can traffic signalling proteins (Roy and Kornberg, 2015).

*T. vaginalis* is a single-celled parasite which colonizes the human urogenital tract and adheres to epithelial cells. Strains that are highly adherent can form clumps more easily than other strains when cultured, and it has been shown that these strains are more cytotoxic to host cells (Coceres et al., 2015; Lustig et al., 2013). Through live imaging, Salas et al. showed that cytonemes are visible on the surface of *T. vaginalis*, and that highly adherent strains have more cytonemes than poorly adherent strains. The number of parasites displaying cytonemes also increased when clumps formed, with the individual parasites within the clumps being connected by cytonemes.

In elegant experiments using inserts with a porous membrane that prevents direct contact between parasites – but allows secreted factors through – Salas et al. demonstrate that extracellular vesicles of less adherent strains stimulate the growth of cytonemes in the most adherent strain. Moreover, overexpressing the protein VPS32 – which regulates the biogenesis of small vesicles – stimulated the induction of cytonemes, suggesting their formation is, in part, contact-independent.

These findings raised the question of what signals within extracellular vesicles from poorly adherent strains stimulate cytoneme formation in highly adherent strains. To investigate, Salas et al. compared the proteins expressed in the extracellular vesicles of multiple strains of *T. vaginalis*, finding differences in protein expression but conservation of the biological processes they are involved in. The strains shared essential components of metabolic processes, signal response, development and locomotion. Furthermore, all strains contained proteins associated with the formation of cytonemes.

Finally, Salas et al. harnessed the ability of *T. vaginalis* to change from a swimming flagellate form to an amoeboid when it adheres to prostate cells, in order to investigate how communication impacts its behavior during infection (Kusdian et al., 2013). Again, through controlled experiments using porous inserts, they showed that less adherent strains induce the amoeboid morphology and the adhesion of the highly adherent strain to prostate cells. Surprisingly, this parasite-to-parasite communication also doubled the adhesion of a poorly adherent strain to the cells.

The experiments provide solid evidence of the participation of extracellular vesicles in communications between parasites, as well as the presence of specific membranous structures that allow this communication. The finding that parasites of different strains communicate with one another raises fundamental questions related to parasitism and the pathology of *Trichomonas*. Why do poorly adherent strains have a greater effect on the formation of cytonemes by adherent strains than their own strain? Do the secreted extracellular vesicles signal the presence of another strain, alerting nearby parasites to enhance their adherence in order to outcompete competitors? Future work should also investigate the role of microbiota and infections that often occur alongside *Trichomonas*, such as *Mycoplasma* (Margarita et al., 2020), in parasite communication and behavior.

**Figure 1.**
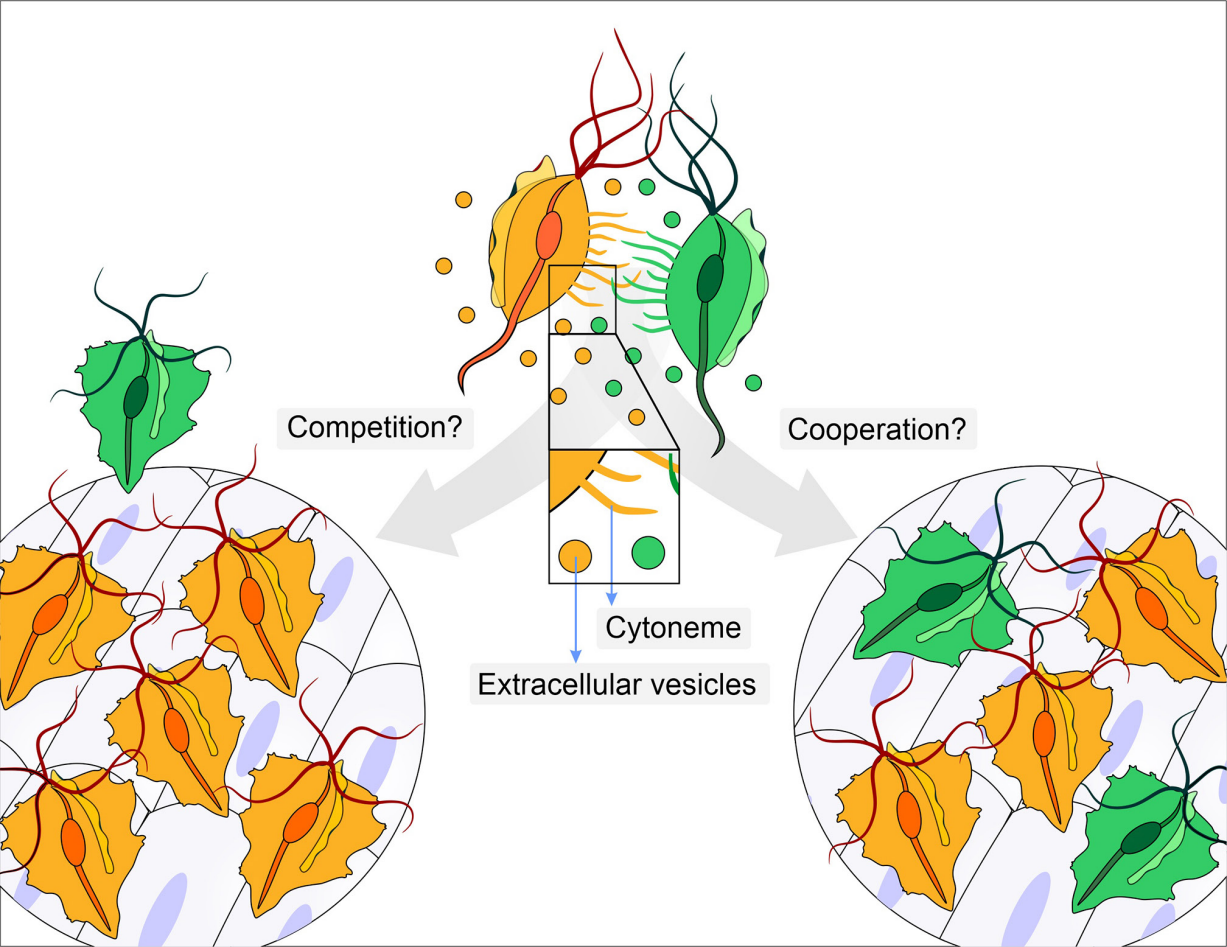
Does parasite communication result in competition or cooperation? Different strains of *Trichomonas vaginalis* (yellow and green) communicate with one another through the release of extracellular vesicles and the formation of membrane protrusions called cytonemes (depicted in inset). This communication can lead to increased adherence of the parasites to epithelial cells. It is not clear whether this communication leads to competition (left), where one strain (yellow) enhances its adherence in order to outcompete a less adherent strain (green), or whether it leads to cooperation (right) where a less adherent strain (green) becomes more adherent after contact with a highly adherent strain (yellow).
